# The role of polymorphisms in ADAM33, a disintegrin and metalloprotease 33, in childhood asthma and lung function in two German populations

**DOI:** 10.1186/1465-9921-7-91

**Published:** 2006-06-19

**Authors:** Michaela Schedel, Martin Depner, Carola Schoen, Stephan K Weiland, Christian Vogelberg, Bodo Niggemann, Susanne Lau, Thomas Illig, Norman Klopp, Ulrich Wahn, Erika von Mutius, Renate Nickel, Michael Kabesch

**Affiliations:** 1 University Children's Hospital, Ludwig Maximilian's University Munich, Germany; 2 Department of Epidemiology, University of Ulm, Germany; 3 University Children's Hospital Dresden, Germany; 4 Department of Pediatric Pneumology and Immunology, Charité Humbolt University Berlin, Germany; 5 Institute of Epidemiology, GSF -Research Centre for Environment and Health, Neuherberg, Germany

## Abstract

**Background:**

*ADAM33*, the first asthma candidate gene identified by positional cloning, may be associated with childhood asthma, lung function decline and bronchial hyperresponsiveness. However, replication results have been inconclusive in smaller previous study populations probably due to inconsistencies in asthma phenotypes or yet unknown environmental influences. Thus, we tried to further elucidate the role of *ADAM33 *polymorphisms (SNPs) in a genetic analysis of German case control and longitudinal populations.

**Methods:**

Using MALDI-TOF, ten *ADAM33 *SNPs were genotyped in 1,872 children from the International Study of Asthma and Allergy in Childhood (ISAAC II) in a case control setting and further 824 children from the longitudinal cohort Multicentre Study of Allergy (MAS). In both populations the effects of single SNPs and haplotypes were studied and a gene environment analysis with passive smoke exposure was performed using SAS/Genetics.

**Results:**

No single SNP showed a significant association with doctor's diagnosis of asthma. A trend for somewhat more profound effects of *ADAM33 *SNPs was observed in individuals with asthma and BHR. Haplotype analyses suggested a minor effect of the *ADAM33 *haplotype H4 on asthma (p = 0.033) but not on BHR. Associations with non atopic asthma and baseline lung function were identified but no interaction with passive smoke exposure could be detected.

**Conclusion:**

The originally reported association between *ADAM33 *polymorphisms and asthma and BHR could not be confirmed. However, our data may suggest a complex role of *ADAM33 *polymorphisms in asthma ethiology, especially in non atopic asthma.

## Background

*ADAM33*, a disintegrin and metalloproteinase 33 has been the first gene published, which had been identified by positional cloning as a putative candidate gene for the development of asthma and bronchial hyperresponsiveness [1]. It has been speculated that the *ADAM33 *gene, expressed in airway smooth muscle cells and fibroblasts of the lung, codes for a protein important for cell fusion, cell adhesion, cell signalling and proteolysis. Furthermore, ADAM33 was suggested to play a role in airway remodeling [2].

The *ADAM33 *gene is located on chromosome 20p13 and 37 SNPs have initially been identified [1]. Ever since the first report of association between *ADAM33 *polymorphisms and asthma in two Caucasian populations from the UK and the USA, a number of replication studies have been published with very diverse results. Various associations between different asthma phenotypes as well as with BHR and several different SNPs in the gene have been reported [3-7]. One possible explanation for this diversity in replication results could be the heterogeneity between study populations or between the definitions of asthma in different study populations. It can be hypothesized that ADAM33, involved in remodeling, may be especially important in some specific forms of asthma. Thus, it may be more relevant in adult or non-atopic than in atopic asthma. Furthermore, it may affect lung function more than atopy status. Finally, environmental factors such as passive smoke exposure could potentially interact with ADAM33 in exerting its remodeling function in the lung as ADAM33 also seems to be involved in COPD mediated processes[8].

**Figure 1:**
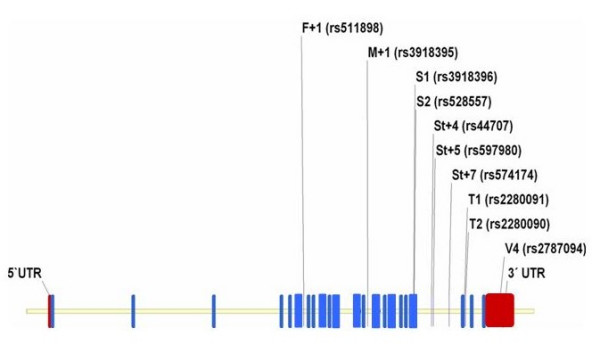
**Position of the genotyped polymorphisms (SNPs) in the ADAM33 gene in respect to the 22 exons (blue) and untranslated regions (red) of the gene**. SNPs nomenclature according to the initial report by Van Eerdewegh et al. and alternatively according to the rs system in brackets.

We tested the hypothesis that the *ADAM33 *gene is associated with atopic or non atopic asthma, lung function and BHR in a large nested case control study of German children (N = 1,872; comparing 624 asthmatics and/or BHR positives and 1,248 non-asthmatic, BHR negative, non-atopic controls) and a German multicentre family based birth cohort study (MAS) (888 children with DNA available, 96 asthmatics and 792 non-asthmatics). The effect of ten SNPs spanning the ADAM33 gene as indicated in figure 1, previously showing associations with asthma phenotypes in some populations, and haplotypes of these SNPs were analysed.

## Methods

### Description of the case control study population

Between 1995 and 1996, cross sectional studies were conducted in Munich (ISAAC II), Dresden (ISAAC II) and Leipzig to assess the prevalence of asthma and allergies in schoolchildren age 9 to 11 years [9,10]. As the populations and phenotyping methods have been described in detail before [9], only an overview of the methods pertaining to this analysis is given here. Parental questionnaires for self-completion were sent through the schools to the families including the ISAAC core questions while slightly different questionnaires were used for the Leipzig population [9]. All children in the three cities whose parents reported that a doctor diagnosed "asthma" at least once or "asthmatic, spastic or obstructive bronchitis" more than once were defined as having asthma.

In Dresden and Munich, children underwent skin prick testing for six common aeroallergens (*Dermatophagoides pteronyssinus, D. farinae*, *Alternaria tenuis*, cat dander, and mixed grass and tree pollen) while in Leipzig *D. pteronyssinus*, grass, birch and hazel pollen, cat and dog dander was examined [10]. A positive skin reaction was defined as a wheal size ≥ 3 mm after subtraction of the negative control [9].

In the Munich and Dresden population, standard baseline lung function was measured and bronchial reactivity was assessed in a random 50% sub-sample of the study population by inhalation of nebulized, hyperosmolar saline (4.5%). Children with a drop in FEV_1 _of 15% or more from baseline were classified as positive for bronchial hyperresponsiveness [9]. In the Leipzig population, measurements of airway challenges utilizing cold-air challenge were performed according to a previously published protocol [10]. In this case BHR was defined as a fall in FEV_1 _of 9% corresponding to a value as large or larger than the 95th percentile of the reference population [11].

For this analysis, all children of German origin who had both DNA and IgE data available and had a doctor's diagnosis of asthma and/or showed BHR (N = 624, Munich n = 230, Dresden n = 263, Leipzig n = 131) were selected from the total study population. These children were matched at a 1:2 ratio with a random selection of healthy, non asthmatic, non atopic children without a diagnosis of BHR (Munich n = 460, Dresden n = 526, Leipzig n = 262) in the analysis.

### Multicentre Allergy Study cohort

The German Multicentre Allergy Study (MAS) cohort has been described in detail elsewhere [12,13]. Initially, 1,314 children born in five German cities in the year 1990 were followed up from birth to the age of 13 years. For 888 children DNA was available and of these, only children of German origin were included in this study (n = 824). Yearly follow-up visits included standardized interviews, questionnaires, and physical examinations. In the MAS study, asthma, hay fever and atopic dermatitis at age 10 were defined using the ISAAC-core questions for children as described for the ISAAC study population.

Serum samples were obtained from the children at birth, and at 1, 2, 3, 5, 6, 7 and 10 years of age. Total IgE, specific IgE antibodies to food allergens and inhalant allergens (*Dermatophagoides pteronyssinus*, cat dander, mixed grass, birch pollen, as well as dog dander from age 3 years on) were determined by CAP-RAST FEIA (Pharmacia & Upjohn, Freiburg, Germany). In the MAS study, atopy was defined as a specific IgE level (CAP I) of ≥ 0.35 kU/l at age 7 or 10 years, respectively.

While pulmonary function tests were performed at age 7, 10 and 13, bronchial hyperresponsiveness was only assessed at age 7 in 610 individuals [14]. Bronchial challenges in the MAS study were conducted after baseline spirometry using increasing concentrations of histamine (usually from 0.5 mg/ml to 8.0 mg/ml) according to standard procedures. The 90^th ^percentile of the distribution of PC_20_FEV_1 _in a healthy subsample corresponded to 0.85 mg/ml. Bronchial hyperresponsiveness was defined as a PC_20_FEV_1 _greater than this value.

Current environmental smoke exposure was defined as any current environmental tobacco smoke exposure at the age of the survey in ISAAC (9–11) and at the age 10-survey in MAS according to the information derived from parental questionnaires. *In utero *exposure to maternal smoking was assessed by a positive answer to the question "Did the mother of the child smoke during pregnancy?". Informed written consent was obtained from all parents of children included in the ISAAC and MAS studies. All study methods were approved by the local ethics committees.

### Genotyping methods

For genotyping, the MassARRAY system (Sequenom, San Diego, USA) was used as previously described in detail [15]. All PCR reactions were performed using standard thermocyclers (MJ Research, Waltham, USA). First, a PCR was carried out. To remove excessive dNTPs, shrimp alkaline phosphatase was added to the PCR products. The base-specific extension reaction was performed in 10 μl reactions by Thermosequenase (Amersham, Piscataway, USA). For the base extension reaction the denaturation was performed at 94°C for 2 min, followed by 94°C for 5 sec, 52°C for 5 sec, and 72°C for 10 sec for 55 cycles. The final base extension products were treated with SpectroCLEAN resin to remove salts out of the reaction buffer, and 16 μl of water was added into each base extension reaction. After a quick centrifugation (2,000 rpm, 3 min) the reaction solution was dispensed onto a 384 format SpectroCHIP pre-spotted with a matrix of 3-hydroxypicolinic acid (3-HPA) by using a SpectroPoint nanodispenser. A modified Bruker Biflex matrix assisted laser desorption ionization-time-of-flight mass spectrometer was used for data acquisitions from the SpectroCHIP. Genotyping calls were made in real time with MASSARRAY RT software (Sequenom, San Diego, USA).

### Statistical analysis

Deviations from Hardy-Weinberg equilibrium were investigated for all polymorphisms using the χ^2 ^statistic, with expected frequencies derived from allele frequencies. Associations between SNPs and qualitative outcomes were determined using Cochran-Armitage-Trend-tests and χ^2^-tests in dominant models of the rare allele. Differences in lung function parameters were tested by univariate variance analysis and t-tests in dominant or recessive models.

Linkage Disequilibrium (LD) and the LD block structure were assessed using Haploview [16] and haplotype analysis was performed for all tagging SNPs after haplotype frequencies had been estimated by the EM (expectation-maximisation) algorithm [17]. Haplotype associations with asthma and BHR were calculated with the haplotype procedure in SAS/Genetics. In addition, haplotype trend regression models were estimated, where the estimated probabilities of the haplotypes were modelled in a logistic regression as independent variables [18].

For asthma as a binary outcome, logistic regression models for gene-environment interactions were used to estimate the combined effect of each SNP with exposure to environmental tobacco smoke (in utero and at time of survey). A Botto Khoury approach summarizing the data in a 2 × 4 table allowed for the evaluation of the independent and combined roles of genotype and exposure on disease risk [19].

All statistical analyses were carried out using the SAS statistical software package (Version 9.1) and the SAS/Genetics module.

## Results

Ten polymorphisms previously identified and showing associations in at least one replication study were selected for genotyping (table 1). These SNPs, located in the 3' half of the gene, spanned the known *ADAM33 *linkage structure as indicated in figure 1. Call rates for SNPs in *ADAM33 *ranged from 90.9% to 93.7% in the family based study population, from 92.1% to 94.3% in the case control population and from 91.9% to 94.0% in the pooled sample as indicated in table 1. All SNPs were in Hardy Weinberg equilibrium in both populations. In the population based cross sectional study populations from East and West Germany, all SNPs showed allele frequencies similar to those previously published in other Caucasian populations (table 1). Genotype frequencies and linkage disequilibrium were almost identical in both the case control population and the MAS cohort (data not shown).

**Table 1:** Description of the investigated ADAM33 SNPs and assay conditions in the case control and cohort study population.

**position in original publication**	**rs numbers**	**Alleles**	**Minor Allele Frequency**		**PCR Primer**	**Extension Primer**	**Callrate (%)**
			*Case Control*	*Cohort based*			
**F+1**	rs511898	G/A	0.38	0.36	fwd ACGTTGGATGAAAATACTGGGACTCGAGGC rev ACGTTGGATGTGCTGTATCTATAGCCCTCC	ACTCGAGGCCTGTGAATTCC	93.73
**M+1**	rs3918395	G/T	0.15	0.13	fwd ACGTTGGATGGGGCACCAATTAACTAAGGC rev ACGTTGGATGTGAGGGCATGGAAGGTTCAG	GCCGGCTCCCAAGCTCC	92.03
**S1**	rs3918396	G/A	0.09	0.09	fwd ACGTTGGATGAGTCGGTAGCAACACCAGGC rev ACGTTGGATGAATCCCCGCAGACCATGACAC	CCTGCTGGCCATGCTCCTCAGC	92.99
**S2**	rs528557	G/C	0.28	0.28	fwd ACGTTGGATGAGTCGGTAGCAACACCAGG rev ACGTTGGATGACCATGACACCTTCCTGCTG	GCTGCCTCTGCTCCCAGG	91.88
**ST+4**	rs44707	A/C	0.41	0.41	fwd ACGTTGGATGGGAGTGAAAAGATGTGCTGG rev ACGTTGGATGCCACTTCCTCTGCACAAATC	ACAAATCACCTCTGTCACCC	92.58
**ST+5**	rs597980	C/T	0.44	0.45	fwd ACGTTGGATGAGAGAACTGGGTTAAGGCAG rev ACGTTGGATGCCAGCACATCTTTTCACTCC	ACTCCATACCACTGGTCAGCTG	93.81
**ST+7**	rs574174	G/A	0.19	0.19	fwd ACGTTGGATGCTGCCCTTGATGATTCCAAG rev ACGTTGGATGGGAACATCACAGGAAATGAC	ACTGTCCCCATCCCATC	93.66
**T1**	rs2280091	T/C	0.16	0.14	fwd ACGTTGGATGTTCCCTTCTCCCTTCCCTCTC rev ACGTTGGATGTTGCTCAGCCCCAAAGATGG	GGGCGGCGTTCACCCCA	93.77
**T2**	rs2280090	C/T	0.16	0.14	fwd ACGTTGGATGTTCCCTTCTCCCTTCCCTCTC rev ACGTTGGATGTTGCTCAGCCCCAAAGATGG	CCCCACAGCCACTGGACAG	93.95
**V4**	rs2787094	C/G	0.22	0.23	fwd ACGTTGGATGAGAAACAGGAAGGAAGGTCC rev ACGTTGGATGTATGGTTCGACTGAGTCCAC	CTGAGTCCACACTCCCCTG	93.84

### Single SNP analyses with qualitative traits

Associations between *ADAM33 *polymorphisms and the phenotypes asthma and BHR were investigated in both populations. As children in the case control sample were 9–11 years old at the time of disease status assessment and children in the longitudinal MAS study population were assessed using the same ISAAC core questions at age 10, all analyses of association with asthma in the MAS population were also performed at this and no other age. As BHR values were only available at age 7 but not at age 10 in the MAS population, BHR was analyzed separately in both populations.

No significant association could be detected between any tested SNP and doctor's diagnosed asthma, neither in the case control population nor in the cohort study, nor in the pooled dataset (table 2). However, the risk to develop non atopic asthma (as defined as a doctor's diagnosis of asthma in the absence of a positive skin prick test) was increased in carriers of the polymorphic A allele in S1 (OR 1.53, 95%CI 1.01–2.31, p = 0.042) and in carriers of the polymorphic G allele in V4 (OR 1.44, 95%CI 1.03–2.01, p = 0.031). Furthermore, the risk for non atopic asthma was decreased in carriers of the polymorphic T allele for M+1 (OR = 0.60, 95%C.I. 0.40 – 0.91, p = 0.016).

**Table 2:** Odds ratios (OR) and 95% confidence intervals (95% CI) for the association between single *ADAM33 *polymorphisms and asthma and BHR in the case-control population (age 9–11) and in the cohort study population assessed at age ten for asthma and age seven for BHR.

	**Asthma**			**BHR^1^**		**Asthma and BHR^2^**
**SNP**	case-control study	cohort study	pooled	case-control study	cohort study	case-control study

***F+1***	0.92 (0.72–1.17) p = 0.502	0.94 (0.58–1.52) p = 0.795	0.92 (0.74–1.15) p = 0.478	1.27 (0.96–1.69) p = 0.095	1.01 (0.63–1.61) p = 0.962	1.61 (0.90–2.88) p = 0.105
***M+1***	0.86 (0.65–1.13) p = 0.269	0.94 (0.53–1.65) p = 0.819	0.89 (0.70–1.14) p = 0.351	1.18 (0.88–1.59) p = 0.274	1.03 (0.61–1.74) p = 0.919	1.46 (0.84–2.54) p = 0.177
***S1***	1.23 (0.90–1.69) p = 0.201	1.16 (0.64–2.10) p = 0.635	1.20 (0.90–1.58) p = 0.210	1.27 (0.90–1.81) p = 0.175	1.19 (0.67–2.14) p = 0.552	1.10 (0.54–2.25) p = 0.789
***S2***	0.99 (0.78–1.26) p = 0.949	0.97 (0.60–1.56) p = 0.893	0.99 (0.80–1.22) p = 0.908	1.29 (0.98–1.69) p = 0.073	1.00 (0.63–1.59) p = 0.989	1.72 (0.99–3.00) p = 0.052
***ST+4***	1.04 (0.81–1.34) p = 0.766	1.31 (0.79–2.18) p = 0.292	1.10 (0.87–1.37) p = 0.432	0.99 (0.74–1.32) p = 0.956	1.08 (0.67–1.74) p = 0.762	1.22 (0.69–2.18) p = 0.490
***ST+5***	0.94 (0.73–1.22) p = 0.649	0.80 (0.48–1.33) p = 0.384	0.89 (0.71–1.12) p = 0.338	0.95 (0.71–1.28) p = 0.748	1.20 (0.71–2.00) p = 0.495	0.91 (0.51–1.60) p = 0.731
***ST+7***	1.11 (0.86–1.42) p = 0.428	1.16 (0.71–1.89) p = 0.549	1.10 (0.88–1.38) p = 0.384	1.20 (0.90–1.59) p = 0.209	1.15 (0.72–1.84) p = 0.562	0.96 (0.55–1.70) p = 0.901
***T1***	0.96 (0.74–1.25) p = 0.762	0.92 (0.53–1.60) p = 0.764	0.97 (0.76–1.23) p = 0.804	1.20 (0.90–1.61) p = 0.213	1.12 (0.67–1.88) p = 0.662	1.50 (0.87–2.59) p = 0.141
***T2***	0.95 (0.73–1.24) p = 0.711	0.88 (0.50–1.55) p = 0.666	0.96 (0.76–1.22) p = 0.731	1.21 (0.91–1.62) p = 0.196	1.14 (0.68–1.91) p = 0.622	1.51 (0.88–2.61) p = 0.134
***V4***	1.22 (0.95–1.55) p = 0.115	0.82 (0.51–1.34) p = 0.439	1.11 (0.90–1.38) p = 0.329	1.13 (0.85–1.49) p = 0.402	1.13 (0.71–1.78) p = 0.608	1.31 (0.76–2.26) p = 0.332

^1^No pooled analysis because of different techniques of BHR assessment in the case-control study and in the cohort study

^2 ^No analysis in the cohort study because of low number of cases

No significant association between *ADAM33 *polymorphisms and BHR, assessed by histamine challenge in the MAS population at age 7 or with hypertonine saline inhalation or cold air challenge in the case control population at age 9–11, was observed as shown in table 2. As the initial study by van Eerdewegh et al. [1] suggested the major effect of *ADAM33 *polymorphisms in individuals with asthma and concomitant BHR, we investigated this specific phenotype in the case control population. Again, no SNP reached statistical significance in the association analysis (table 2). As BHR values were only available at age 7 but not at age 10 in the MAS cohort study and different procedures were used to define BHR in both study populations, no combined analysis with both outcome variables was performed.

### Single SNP analyses with lung function measurements

Next, the effects of *ADAM33 *SNPs on baseline lung function measurements (FVC, FEV1, MEF25, MEF50 and MEF75) were investigated in cases (asthma and/or BHR positive) and controls separately (table 3). In cases, FVC was increased in carriers of S2, T1 and T2 polymorphisms while FEV1 was increased in carriers of S2 and M+1 polymorphisms. In contrast to polymorphism S1, the presence of the polymorphic C allele in S2 increased the values for MEF75. In controls, negative effects on MEF50 were observed with ST+5 and MEF75 was increased in carriers of the M+1 or S2 SNP.

**Table 3a:** Lung function parameters in the case-control study for all cases *)

**SNP**		**N**^1)^	**MEF_25 _(%) Mean ± SD**	**MEF_50 _(%) Mean ± SD**	**MEF_75 _(%) Mean ± SD**	**FEV1 (%) Mean ± SD**	**FVC (%) Mean ± SD**
***F+1***	wild type	165	89.82 ± 29.66	89.47 ± 22.54	91.90 ± 16.28	97.65 ± 11.44	97.64 ± 11.03
	heterozygous	232	91.86 ± 32.06	91.22 ± 22.23	94.48 ± 18.53	99.32 ± 10.57	99.80 ± 10.02
	homozygous	64	92.61 ± 23.45	90.28 ± 17.19	97.32 ± 18.00	98.56 ± 10.75	98.16 ± 10.75
***M+1***	wild type	317	90.75 ± 30.97	89.70 ± 22.29	93.19 ± 17.82	98.17 ± 11.43*^r^	98.12 ± 11.05
	heterozygous	128	91.28 ± 28.43	91.51 ± 22.15	95.56 ± 17.50	**99.01 ± 9.86**	99.86 ± 9.83
	homozygous	4	109.34 ± 23.03	98.95 ± 12.38	95.75 ± 23.07	**109.31 ± 9.13**	105.89 ± 9.71
***S1***	wild type	359	90.57 ± 30.08	90.00 ± 21.74	93.23 ± 17.00*^v^	98.29 ± 10.86	98.46 ± 10.72
	heterozygous	86	94.29 ± 31.08	92.01 ± 24.43	**97.23 ± 21.03**	99.63 ± 11.23	99.58 ± 10.02
	homozygous	4	73.47 ± 16.07	76.17 ± 10.97	**78.46 ± 11.72**	90.84 ± 5.11	96.06 ± 1.40
***S2***	wild type	215	91.22 ± 31.50	89.69 ± 22.15	92.14 ± 17.03*^d^	97.50 ± 11.49*^d^	96.97 ± 11.32*^v^*^d^
	heterozygous	202	91.30 ± 29.78	91.43 ± 22.16	**96.12 ± 18.27**	**99.76 ± 10.73**	**100.55 ± 9.99**
	homozygous	31	95.31 ± 21.52	90.93 ± 22.04	**94.54 ± 20.57**	**99.55 ± 9.27**	**98.70 ± 9.25**
***ST+4***	wild type	158	90.05 ± 29.28	89.70 ± 22.41	93.18 ± 18.52	98.08 ± 10.38	99.09 ± 10.01
	heterozygous	223	92.00 ± 29.50	91.32 ± 22.23	94.83 ± 18.14	99.06 ± 11.75	98.61 ± 11.11
	homozygous	65	92.97 ± 34.91	90.22 ± 22.04	93.93 ± 14.12	98.68 ± 10.03	98.59 ± 11.39
***ST+5***	wild type	136	92.47 ± 31.51	91.10 ± 20.52	94.37 ± 15.25	99.26 ± 10.00	99.38 ± 10.88
	heterozygous	236	91.87 ± 29.26	91.02 ± 23.37	94.65 ± 19.04	98.79 ± 11.61	98.62 ± 10.58
	homozygous	88	87.50 ± 29.64	87.41 ± 20.55	91.39 ± 17.77	97.13 ± 10.41	98.48 ± 10.15
***ST+7***	wild type	285	90.28 ± 29.48	90.36 ± 21.92	93.30 ± 16.83	98.67 ± 10.93	99.10 ± 10.49
	heterozygous	158	94.26 ± 31.56	91.07 ± 22.55	94.99 ± 19.60	98.98 ± 11.11	98.63 ± 10.92
	homozygous	16	82.30 ± 24.76	82.94 ± 17.26	95.38 ± 16.31	93.87 ± 9.08	95.04 ± 7.62
***T1***	wild type	315	90.42 ± 31.04	89.55 ± 22.40	93.32 ± 17.78	98.07 ± 11.38	98.12 ± 10.83*^d^
	heterozygous	135	92.59 ± 28.08	92.22 ± 21.39	95.50 ± 17.87	99.42 ± 9.78	**100.10 ± 9.98**
	homozygous	8	98.09 ± 27.48	90.95 ± 23.47	91.61 ± 19.64	103.91 ± 11.37	**103.30 ± 8.53**
***T2***	wild type	317	90.60 ± 31.04	89.61 ± 22.34	93.37 ± 17.71	98.13 ± 11.36	98.14 ± 10.80*^d^
	heterozygous	135	92.59 ± 28.08	92.22 ± 21.39	95.50 ± 17.87	99.42 ± 9.78	**100.10 ± 9.98**
	homozygous	8	98.09 ± 27.48	90.95 ± 23.47	91.61 ± 19.64	103.91 ± 11.37	**103.30 ± 8.53**
***V4***	wild type	267	91.58 ± 30.15	89.98 ± 21.82	93.12 ± 17.14	98.24 ± 11.04	98.22 ± 10.82
	heterozygous	163	89.70 ± 30.05	89.71 ± 22.19	94.63 ± 18.60	98.57 ± 10.65	99.20 ± 10.23
	homozygous	26	95.52 ± 30.38	97.28 ± 24.60	96.90 ± 19.51	101.03 ± 11.93	99.82 ± 10.29

*) significant differences (p < 0.05) in lung function parameters between genotypes are printed in bold letters,

*^v ^denotes significant differences in variance analysis,

*^d ^denotes significant differences in t-test for a dominant model,

*^r ^significant differences in t-test for a recessive model

^1)^N refers to the first lung function parameter. Minimal deviations of N in the other lung function parameters are possible.

**Table 3b:** Lung function parameters in the case-control study for all controls *)

**SNP**		**N**^1)^	**MEF_25 _(%) Mean ± SD**	**MEF_50 _(%) Mean ± SD**	**MEF_75 _(%) Mean ± SD**	**FEV1 (%) Mean ± SD**	**FVC (%) Mean ± SD**
***F+1***	wild type	291	98.48 ± 25.83	97.93 ± 19.80	99.22 ± 17.82	100.59 ± 9.86	98.28 ± 10.49
	heterozygous	348	101.46 ± 30.46	99.08 ± 21.41	100.69 ± 17.83	101.76 ± 10.29	99.08 ± 10.26
	homozygous	111	99.44 ± 29.96	99.63 ± 20.23	101.52 ± 19.80	101.38 ± 10.75	98.47 ± 10.38
***M+1***	wild type	522	99.25 ± 27.06	98.22 ± 20.12	99.46 ± 17.92*^d^	101.06 ± 10.11	98.46 ± 10.51
	heterozygous	201	101.97 ± 32.25	100.01 ± 21.94	**102.51 ± 18.60**	102.10 ± 10.69	99.39 ± 10.29
	homozygous	12	95.45 ± 35.80	104.74 ± 19.55	**104.98 ± 13.31**	98.63 ± 8.56	96.65 ± 8.30
***S1***	wild type	627	100.12 ± 29.00	99.05 ± 20.58	100.58 ± 17.89	101.18 ± 10.28	98.67 ± 10.52
	heterozygous	116	98.23 ± 27.90	97.01 ± 21.40	99.16 ± 19.84	101.78 ± 10.41	99.29 ± 10.11
	homozygous	6	95.19 ± 21.20	98.62 ± 16.03	103.61 ± 13.45	103.74 ± 10.25	100.29 ± 11.22
***S2***	wild type	381	99.51 ± 26.91	98.44 ± 20.04	98.82 ± 17.78*^d^	100.94 ± 10.08	98.34 ± 10.67
	heterozygous	292	101.59 ± 31.92	99.11 ± 22.33	**101.88 ± 18.68**	102.09 ± 10.56	99.55 ± 10.28
	homozygous	62	97.76 ± 25.55	100.37 ± 16.24	**101.35 ± 16.99**	102.51 ± 9.76	98.97 ± 9.91
***ST+4***	wild type	257	100.02 ± 28.64	98.04 ± 20.98	100.06 ± 17.63	101.58 ± 9.86	99.20 ± 9.75
	heterozygous	365	100.09 ± 28.74	99.28 ± 21.10	101.17 ± 19.05	101.39 ± 10.50	98.55 ± 10.50
	homozygous	114	98.52 ± 28.40	98.83 ± 18.79	98.41 ± 16.62	100.07 ± 10.09	98.18 ± 11.43
***ST+5***	wild type	222	100.79 ± 31.48	101.33 ± 20.02*^d^	101.79 ± 17.87	101.33 ± 10.00	98.98 ± 10.29
	heterozygous	380	99.54 ± 28.10	**97.52 ± 21.27**	99.77 ± 18.20	100.85 ± 10.58	98.12 ± 10.82
	homozygous	148	99.48 ± 25.81	**97.72 ± 19.54**	99.23 ± 18.08	102.19 ± 9.46	99.74 ± 9.19
***ST+7***	wild type	507	100.34 ± 28.71	99.01 ± 20.67	100.26 ± 17.65	101.40 ± 10.17	98.93 ± 10.29
	heterozygous	213	98.76 ± 29.25	98.00 ± 20.58	100.11 ± 19.04	100.65 ± 9.99	98.01 ± 10.35
	homozygous	32	101.56 ± 24.74	98.45 ± 20.33	99.70 ± 19.21	103.37 ± 12.14	100.57 ± 12.16
***T1***	wild type	531	99.34 ± 27.07	98.23 ± 20.15	99.49 ± 18.08	101.07 ± 10.13	98.58 ± 10.53
	heterozygous	205	101.94 ± 32.34	99.58 ± 21.96	101.97 ± 18.39	101.81 ± 10.47	99.12 ± 10.14
	homozygous	15	95.29 ± 31.85	103.19 ± 18.07	101.58 ± 14.14	101.17 ± 9.28	98.59 ± 8.47
***T2***	wild type	532	99.31 ± 27.05	98.23 ± 20.13	99.51 ± 18.07	101.07 ± 10.12	98.57 ± 10.52
	heterozygous	204	102.01 ± 32.41	99.58 ± 22.01	101.92 ± 18.42	101.80 ± 10.49	99.09 ± 10.16
	homozygous	15	95.29 ± 31.85	103.19 ± 18.07	101.58 ± 14.14	101.17 ± 9.28	98.59 ± 8.47
***V4***	wild type	474	100.07 ± 28.66	98.91 ± 21.18	100.74 ± 18.35	101.32 ± 10.45	98.76 ± 10.49
	heterozygous	249	99.87 ± 29.37	98.91 ± 19.75	99.86 ± 17.75	101.62 ± 9.80	99.16 ± 10.07
	homozygous	35	99.31 ± 25.64	96.87 ± 19.30	100.16 ± 19.15	100.29 ± 11.81	96.95 ± 12.34

*) significant differences in lung function parameters between genotypes are printed in bold letters,

*^v ^denotes significant differences in variance analysis,

*^d ^denotes significant differences in t-test for a dominant model,

*^r ^significant differences in t-test for a recessive model

^1)^N refers to the first lung function parameter. Minimal deviations of N in the other lung function parameters are possible.

### Haplotype analysis

In a further step, haplotypes were estimated in both populations for all samples genotyped successfully for at least one *ADAM33 *SNP (1,802 in the case control population and 782 in the MAS cohort) using the EM algorithm. The estimated frequencies of all common *ADAM33 *haplotypes built from the eight SNPs F+1, S1, S2, ST+4, ST+5, ST+7, T1 and V4 and all ten SNPs are presented in table 4. As SNPs M+1, T1 and T2 were in extremely tight linkage disequilibrium, polymorphisms M+1 and T2 contributed no additional information to the haplotype and thus were excluded from the further haplotype building procedure.

**Table 4:** Estimated frequencies of common haplotypes in different German populations

											**Estimated frequencies in the different populations^3^**		
	**F+1**	M+1^2^	**S1**	**S2**	**ST4**	**ST+5**	**ST+7**	**T1**	T2^2^	**V4**	**pooled**	**case-control study**	**cohort study**

**H1**	**G**	G	**G**	**G**	**A**	**T**	**G**	**T**	C	**C**	31.59% (31.62%)	31.42% (31.43%)	32.21% (32.24%)
**H2**	**G**	G	**G**	**G**	**C**	**C**	**G**	**T**	C	**C**	16.63% (16.66%)	16.66% (16.67%)	16.54% (16.62%)
**H3**	**A**	T	**G**	**C**	**A**	**C**	**G**	**C**	T	**C**	14.00% (13.91%)	14.67% (14.67%)	12.50% (12.20%)
**H4**	**G**	G	**G**	**G**	**C**	**C**	**G**	**T**	C	**G**	11.22% (11.20%)	11.02% (11.01%)	11.48% (11.48%)
**H5**	**A**	G	**A**	**C**	**A**	**T**	**A**	**T**	C	**G**	7.84% (7.84%)	7.73% (7.73%)	8.12% (8.12%)
**H6**	**A**	G	**G**	**G**	**C**	**C**	**A**	**T**	C	**C**	6.48% (6.50%)	6.68% (6.70%)	6.06% (6.07%)
**Rare^1^**											12.24% (12.28%)	11.82% (11.80%)	13.10% (13.27%)

^1) ^Rare are all haplotypes with an estimated frequency < 0.03 in the pooled sample

^2) ^SNPs which are excluded after choosing only tagging SNPs

^3) ^Estimated frequencies of the 8-SNP-haplotype, in brackets estimated frequencies of the 10-SNP-haplotype

One common haplotype, H4 (G-G-G-C-C-G-T-G), showed a weak but not significant association with asthma in the case control population but not in the cohort as indicated in table 5a. This association became significant in the pooled analysis. No association was found with BHR (data not shown). When a haplotype trend regression was performed for the haplotype H4, an OR of 1.57 (95%CI 0.99–2.51, p = 0.057) in the pooled population was observed.

**Table 5:** Estimated haplotype frequencies and associations with asthma in case control and cohort populations

	**Haplotype**	**Study population**^1)^	**Haplotype frequencies in the cases**	**Haplotype frequencies in the controls**	**Odds Ratio and Confidence intervals**^2)^	**p-value of χ^2^-Test**
H1	**G-G-G-A-T-G-T-C**	**all**	30.35%	32.21%	0.92(0.78–1.08)	0.293
		**ccs**	30.90%	31.56%	0.97(0.81–1.15)	0.735
		**coh**	30.03%	34.02%	0.83(0.58–1.19)	0.317
H2	**G-G-G-C-C-G-T-C**	**all**	17.02%	16.69%	1.02(0.84–1.25)	0.804
		**ccs**	16.77%	16.85%	0.99(0.80–1.24)	0.939
		**coh**	16.93%	16.32%	1.04(0.67–1.63)	0.847
H3	**A-G-C-A-C-G-C-C**	**all**	12.98%	14.08%	0.91(0.73–1.13)	0.402
		**ccs**	13.60%	14.85%	0.90(0.71–1.15)	0.406
		**coh**	10.33%	12.04%	0.84(0.49–1.45)	0.531
H4	**G-G-G-C-C-G-T-G**	**all**	12.96%	10.44%	1.28(1.02–1.60)	0.033
		**ccs**	13.15%	10.65%	1.27(0.99–1.64)	0.063
		**coh**	11.43%	9.85%	1.18(0.70–2.00)	0.528
H5	**A-A-C-A-T-A-T-G**	**all**	8.35%	7.69%	1.09(0.83–1.43)	0.517
		**ccs**	8.62%	7.16%	1.22(0.90–1.66)	0.191
		**coh**	7.18%	9.07%	0.78(0.41–1.46)	0.430
H6	**A-G-G-C-C-A-T-C**	**all**	6.70%	6.54%	1.03(0.76–1.38)	0.867
		**ccs**	6.17%	6.67%	0.92(0.65–1.30)	0.638
		**coh**	8.99%	6.19%	1.50(0.82–2.72)	0.184

^1) ^All = case control study and cohort study population pooled, ccs = case control study (n = 358 cases/n = 1198 controls), coh = cohort study (n = 82 cases/n = 464 controls); children without haplotype information were excluded

^2) ^Odds Ratios were calculated as one haplotype vs. all others

### Gene environment interaction analysis

As it was hypothesised that ADAM33 could influence the effects of passive smoke exposure on asthma, BHR or lung function, gene environment interactions were assessed using a Botto Khoury approach. However, no such interactions could be detected (data not shown).

## Discussion

We have genotyped 2,696 subjects including more than 700 children with asthma and/or BHR for 10 SNPs in the *ADAM33 *gene. For doctor's diagnosed asthma, no SNP showed a significant association in any of the analyzed populations. A trend for somewhat more profound but not significant effects of *ADAM33 *SNPs was observed in individuals with asthma and BHR, for which trait the most significant association results were reported in the original study on *ADAM33*. However, in the case control population, these associations did just not reach statistical significance. Haplotype analyses suggested a minor effect of the *ADAM33 *haplotype H4 on asthma but not BHR. A number of individual SNPs showed an association with non atopic asthma in the case control population. A diverse picture evolved when the effects of *ADAM33 *polymorphisms on baseline lung function were measured. However, these associations did not remain significant after correction for multiple testing. No interaction with passive smoke exposure could be detected.

*ADAM33 *was the first published candidate gene for asthma identified by positional cloning. In the initial report 37 SNPs in the *ADAM33 *gene have been identified and 15 polymorphisms have been genotyped in a UK and a US study population [1]. Even within these two populations, different SNPs were associated with asthma and BHR. Associations were significantly stronger in those cases additionally showing BHR, suggesting that ADAM33 acts via lung specific mechanisms. A putatively functional role for ADAM33 in the pathogenesis of asthma has been hypothesised as ADAM33 is expressed in smooth muscle cells of the bronchial and vascular system in the lung [1,20]. It has been speculated that ADAM33 may act as a protease activating cytokine or induce airway smooth muscle proliferation. ADAM33 and its so far identified polymorphisms may have less to do with atopic inflammation and more with non atopic lung specific forms of asthma. To a somewhat lesser degree, this initial BHR effect was confirmed in our study population.

In terms of replication on a population level, the role of *ADAM33 *SNPs in asthma remains controversial. It seems that even the studies reporting a positive association between *ADAM33 *SNPs and atopic phenotypes are inhomogeneous in their findings (table 6). These inconsistencies in replication may have different reasons. They could be due to population heterogeneity, as some studies may suggest. Howard and co-workers genotyped 8 SNPs in 4 different ethnical populations (Dutch, white Americans, Hispanics and African Americans) and found a wide variety of associations between the different ethnical groups and various *ADAM33 *SNPs [4]. No single SNP was associated with asthma in all 4 groups and when corrected for multiple testing, only one association remained significant. In studies of asthmatics with a Hispanic background, no association with *ADAM33 *SNPs was observed with asthma [21]. Thus, differences in haplotype structure or even in the occurrence of SNPs may exist between ethnicities, which have not yet been investigated sufficiently for *ADAM33 *but which are known to exist for a number of other genes.

**Table 6:** Comparison of previously reported association results^1 ^with *ADAM33 *polymorphisms

**Study**	**Study population**	**N^2^**	***ADAM33 *SNPs and reported associations**																
	cc = case control fa = family study	cases/controls families (ind.)	**F+1**	**I1**	**L-1**	**M+1**	**Q-1**	**S1**	**S2**	**ST+1**	**ST+4**	**ST+5**	**ST+7**	**T1**	**T2**	**T+1**	**V-1**	**V1**	**V4**

**Van Eerdewegh et al**.	US/UK combined (cc)	130/217	neg	neg	neg	neg	A	A	neg	neg	A	neg	A	neg	neg	neg	A	neg	A
	UK (cc)	(not reported)	A	neg	neg	neg	A	A	A	neg	A	neg	neg	neg	neg	neg	A	neg	A
	US (cc)	(not reported)	neg	A	A	A	neg	neg	neg	neg	neg	neg	neg	A	A	A	neg	neg	neg
	US/UK (fa)	460fa (1840)						A						B			neg	neg	neg

**Lind et al**.	Mexican (cc)	190/160						neg						neg	neg		neg	neg	neg
	Puerto Rican (cc)	183/165						neg						neg	neg		neg	neg	neg
	Mexican/P. Rican (fa)	583fa (1749)^3^						neg						neg	neg		neg	neg	neg

**Raby et al.^4^**	Non-Hispanic white (fa)	474 (1462)				neg			neg		neg	neg		neg	neg	neg	neg		neg
	Hispanic (fa)	47 (149)				neg			neg		neg	neg		A	neg	A	neg		neg
	African American (fa)	66 (203)				neg			neg		neg	neg		neg	neg	neg	neg		neg

**Werner et al**.	German (cc)	48/499	neg	neg		neg	neg	neg	neg	neg	neg	B	A	neg	neg	neg	neg		neg
	German (fa)	171fa (732)	A, B, AB	neg		neg	neg	neg	B, AB	neg	A	A	neg	neg	neg	neg	neg		neg

**Howard et. al**.	African American (cc)	161/265^5^						neg	A		AT		neg	neg	neg		neg		AT
	US White (cc)	220/229^5^						neg	A^6^, AT		neg		A	A, AT	A, AT		neg		neg
	US Hispanic (cc)	113/127^5^						neg	A		AT		neg	A^6^, AT	A, AT		AT		neg
	Dutch White (cc)	180/133^5^						AT	AT		neg		A, AT	neg	neg		AT		A

**Blakey et al**.	Icelandic (cc)	348/262	neg			neg	neg	neg	neg		neg	neg	neg	neg	neg	neg	neg		neg
	Nottingham (fa)	60fa (240)	neg			neg	neg	neg			neg	neg	neg	neg	neg	neg	neg	neg	neg

^1 ^association with asthma (= A), BHR (= B), Asthma and BHR (= AB) or Atopy (= AT) in different studies; SNPs which are not significantly associated = neg

^2 ^in case-control studies number of cases and controls, in family studies number of families and individuals

^3^265 Mexican families and 318 Puerto Rican families, no association neither in single nor in pooled population

^4^in the study of Raby et al. 8 additional SNPs were investigated: G1, I1, KL+3, N1, S+1, T+2, V-2, V3. None of the additional SNPs showed an association with asthma.

^5^N is reported as maximum number of successfully genotyped subjects

^6^only a trend (0.05 < p ≤ 0.06)

A further explanation for the differences in replication results might be that the definition of asthma may have varied between studies. As ADAM33 may specifically affect remodeling of the lungs, the impact of genetic variations in ADAM33 could be variable in different forms of asthma. In other words, *ADAM33 *genetics may have more impact on those forms of asthma which are less driven by atopy and more associated with lung specific mechanisms. Our data indeed suggests that the known *ADAM33 *SNPs have only a minor impact on the most common form of childhood asthma, which is highly correlated with atopy in most study populations. In contrast, a number of *ADAM33 *SNPs were associated with non atopic asthma as well as baseline lung function measurements in our study. However, the pattern of association remains complex as different SNPs are associated with non atopic asthma and determinants of lung function. Moreover, *ADAM33 *SNPs also seem to play a different role in adult asthma than in childhood asthma as indicated by previously published studies. Werner et al. genotyped 15 *ADAM33 *SNPs in a family based and in an adult case control population and observed variable associations between SNPs and asthma within the two populations and also in respect to the initially reported associations [7]. Very large studies investigating childhood asthma by Lind [21] and Raby [6] could not find any association between single *ADAM33 *SNPs or haplotypes and childhood asthma. However, no analyses of *ADAM33 *effects on non atopic asthma have been reported in these studies of childhood asthma. While Raby et al. [6] stated that it seems very unlikely that these negative studies were underpowered to detect an association, a recent meta analysis suggested, that the odds ratio for the ADAM33 locus may be in the order of 1.4 or lower for SNPs known to date [3]. Even with large data sets such as used in this study, the ability to detect risks of this magnitude may be limited. Furthermore, it may be possible that the known SNPs in ADAM33 are only a proxy for additional, yet unidentified SNPs in the ADAM33 gene, which could be the true cause for the observed but mixed signals from this locus.

Finally, differences in the study populations in terms of gene by environment interactions may also explain some of the observed discrepancy in replication as has been suggested to be the case with other genes inconsistently replicated [22,23]. However, it is not clear, which environmental factors may interact with *ADAM33 *genetics and if these factors could influence the associations between *ADAM33 *polymorphisms and asthma. As indicated by our analysis, passive smoke exposure does not seem to be one of these factors.

In the meantime, a total of five genes (*ADAM33*[1], *PHF11*[24], *DPP10 *[25]*GPRA *[26] and *HLA-G *[27] have been proposed as potential asthma genes by positional cloning and some more may follow. What can we learn from the experience with *ADAM33*? First, it seems that genes identified by positional cloning have the same limitations as other putative candidate genes suggested by expression studies, or selected because of their biological context in disease pathways. Positional cloning does not prove but suggest a role of the gene in question for a specific disease. Further evidence however can only be achieved by independent replication studies and functional molecular genetics, which both may be tedious. This process may take some time and the level of evidence for or against the involvement of a certain gene in a complex disease may only increase with time. Large and well defined replications are needed and negative results ought to be published.

## Conclusion

Our data suggest that previously reported *ADAM33 *polymorphisms may only have a minor impact on the development of asthma in German children.

## Abbreviations

ADAM33 a disintegrin and metalloprotease 33

BHR bronchial hyperresponsiveness

EM expectation-maximisation

FEV1 forced expiratory volume in one second

FVC forced vital capacity

IgE immunoglobulin E

ISAAC II International Study of Asthma and Allergy in Childhood

LD linkage disequilibrium

MAS Multicentre Allergy Study

MALDI-TOF Matrix-assisted laser desorption time of flight

MEF maximum expiratory flow

OR odds ratio

PC_20 _Provocative concentration inducing a 20% fall in FEV1

PCR polymerase chain reaction

SNP single nucleotide polymorphism

## Competing interests

The author(s) declare that they have no competing interests.

## Authors' contributions

MS participated in genotyping, data analysis and manuscript preparation; MD performed data analysis and participated in manuscript preparation; CS, NK and TI participated in genotyping, CF, CV, BN and SL collected data; SW, UW, EvM, RN and MK contributed to the development of the study design, collection of data, data analysis, and manuscript preparation.
